# Supplementary material for: Understanding Gel-Powers: Exploring Rheological Marvels of Acrylamide/Sodium Alginate Double-Network Hydrogels
Source: Molecules. 2023 Jun 20;28(12):4868. doi: 10.3390/molecules28124868 (PMC10305142; doi:10.3390/molecules28124868)
Supplement: Supplementary file 1 [file molecules-28-04868-s001.zip › molecules-2453661-supplementary.pdf]

Supporting Information

# Understanding Gel-Powers: Exploring Rheological Marvels of Acrylamide/Sodium Alginate Double-Network Hydrogels

**Shi-Chang Wang [1], Shu-Tong Du [2], Saud Hashmi [3], Shu-Ming Cui [1,4], Ling Li [1], Stephan Handschuh-Wang [2,4], Xuechang Zhou [2] and Florian J. Stadler [1,*]**

[1] College of Materials Science and Engineering, Shenzhen Key Laboratory of Polymer Science and Technology, Guangdong Research Center for Interfacial Engineering of Functional Materials, Shenzhen University, Shenzhen 518055, China
[2] College of Chemistry and Environmental Engineering, Shenzhen University, Shenzhen 518055, China
[3] Department of Polymer & Petrochemical Engineering, NED University of Engineering & Technology, Karachi 75270, Pakistan
[4] The International School of Advanced Materials, School of Emergent Soft Matter, South China University of Technology, Guangzhou 511442, China
* Correspondence: fjstadler@szu.edu.cn

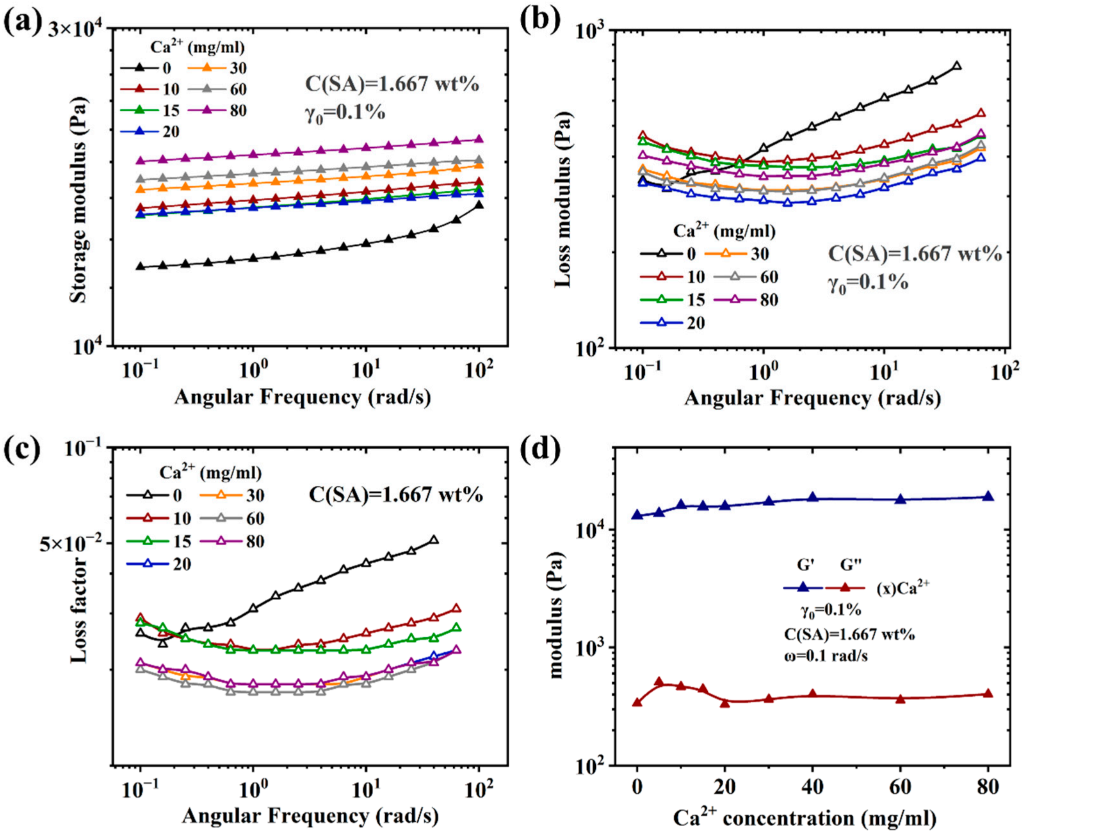


*Figure S1 (a and b) Frequency scan curves for sample S2 (SA=2.667 wt%), (c) relationship between loss coefficient and angular frequency in the frequency scan, (d) modulus of sample S2 versus calcium ion concentration*

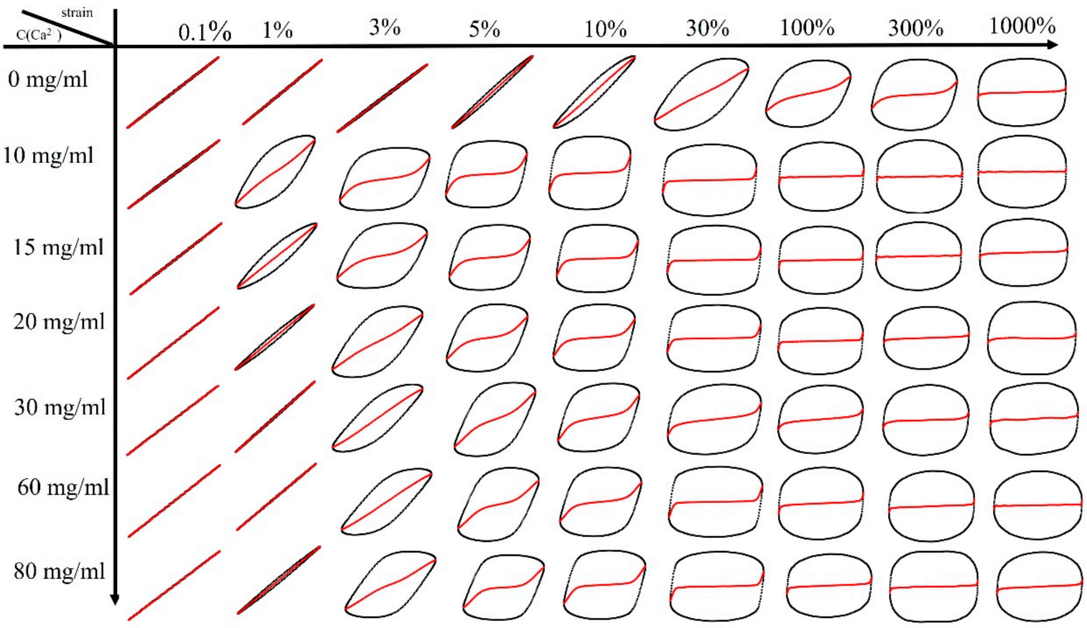


*Figure S2 Elastic Lissajous curves of sample S1 at different calcium ion concentrations with different $\gamma_0$*

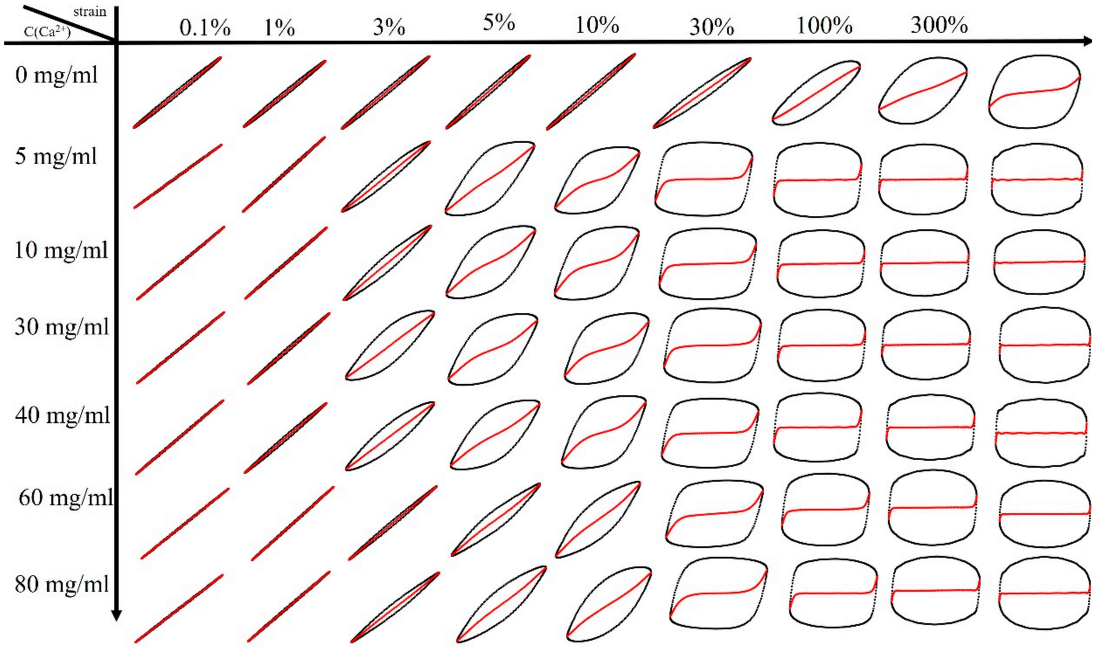


*Figure S3 Elastic Lissajous curves of sample S2 at different calcium ion concentrations and different $\gamma_0$*

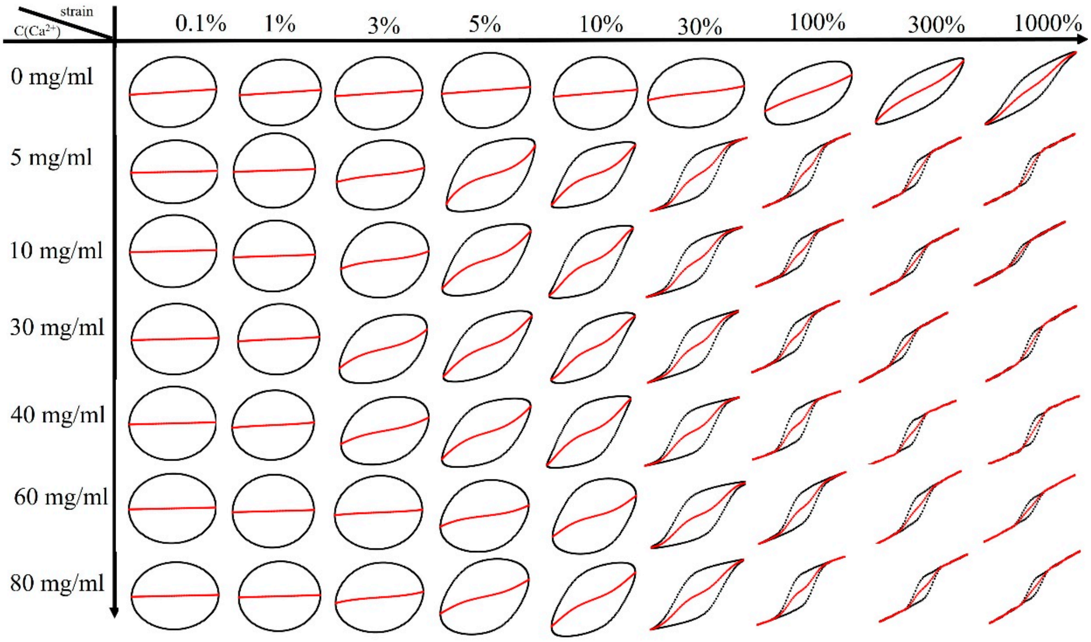


*Figure S4 Viscosity Lissajous curves of sample S2 at different calcium ion concentrations with different $\gamma_0$*

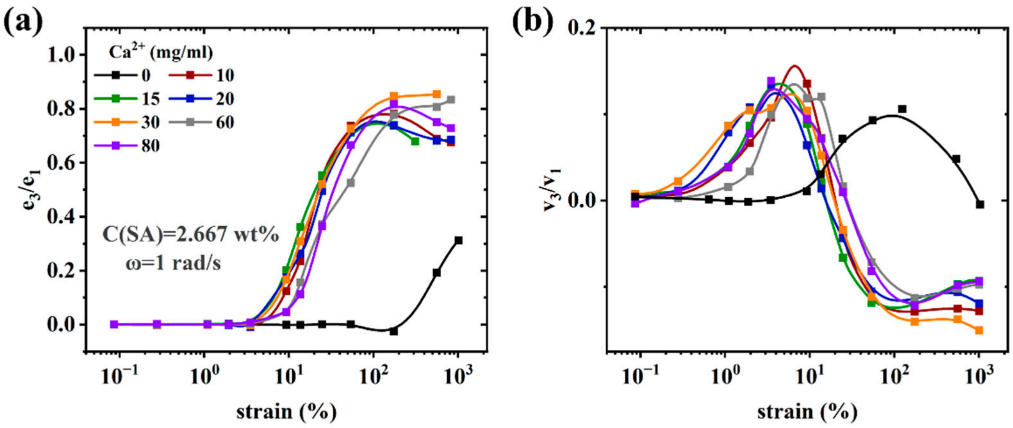


*Figure S5 sample S2: (a and b) Chebyshev coefficient ($e_3/e_1$, and $v_3/v_1$) versus strain*
